# Giving new life to an outdated spectrofluorometer for static and time-resolved UCNP optical characterization†

**DOI:** 10.1039/d5na00330j

**Published:** 2025-05-27

**Authors:** Tomás Di Napoli, Juan M. Bujjamer, Marcos Illescas, Beatriz Barja, Hernán E. Grecco

**Affiliations:** a Universidad de Buenos Aires, Facultad de Ciencias Exactas y Naturales, Departamento de Física Buenos Aires Argentina hgrecco@df.uba.ar; b CONICET - Universidad de Buenos Aires, Instituto de Física de Buenos Aires (IFIBA) Buenos Aires Argentina; c Universidad de Buenos Aires, Facultad de Ciencias Exactas y Naturales, Departamento de Química Inorgánica, Analítica y Química Física Buenos Aires Argentina; d CONICET - Universidad de Buenos Aires, Instituto de Química Física de los Materiales, Medio Ambiente y Energía (INQUIMAE) Buenos Aires Argentina

## Abstract

The obsolescence of proprietary, closed-source software and electronics renders high-quality scientific equipment inoperable, particularly affecting low-income countries where replacement costs hinder research and student training. Institutions often prioritize renewing equipment that addresses the needs of a larger user base, thereby limiting the emergence of research lines, such as up-conversion studies, that require more specific equipment. Refurbishing older equipment with open-source solutions offers a cost-effective way to extend its lifespan while introducing new functionalities. In this work, we present the refurbishment and enhancement of a 30 year-old Horiba PTI QuantaMaster 400 spectrofluorometer, retrofitted to perform not only steady-state, but also time-resolved spectral measurements. We replaced the outdated control system, which relied on proprietary ISA boards and closed-source FelixGX software running on Windows 95, with a modern Red Pitaya (RP) CPU and FPGA board running Linux. We developed a Python application that replicates the original functionality through both a graphical user interface (GUI) and an application programming interface (API). Additional improvements included replacing the monochromator motor driver with DRV8825 integrated circuits controlled by the RP's digital IO, as well as integrating photon counting through the RP's analog inputs. We added a computer controlled infrared laser to enable steady-state and time-resolved spectroscopic measurements of the upconversion process. We demonstrate such extended system capabilities by characterizing β-NaYF_4_:Yb^3+^, Er^3+^ upconversion nanoparticles (UCNPs) in the millisecond range with microsecond resolution. The refurbished instrument now operates with open source software and hardware, offering enhanced functionality, programmability, and long-term sustainability, providing a cost-effective solution for advancing research in resource-limited settings.

## Abstract 2

Obsolescence of proprietary systems can render valuable scientific instruments useless. Here we refurbished and enhanced a spectrofluorometer, enabling full optical characterization of upconversion nanoparticles.

## Graphical abstract

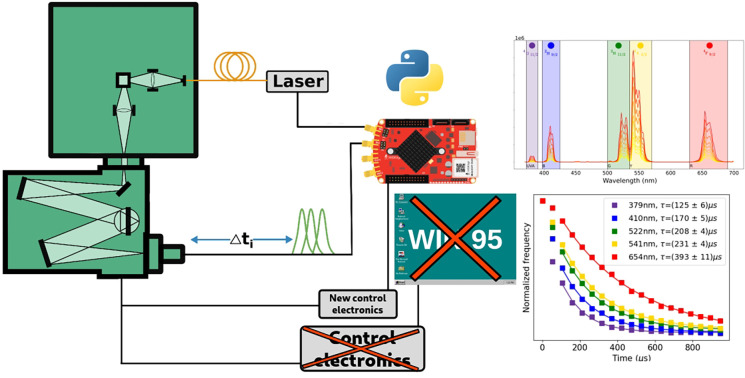

## 1 Introduction

Scientific instruments play a pivotal role in facilitating groundbreaking research and enabling students to acquire hands-on experience in their respective disciplines. In middle and low-income countries, institutions operate with limited financial resources and inadequate infrastructure, further widening disparities in access to advanced scientific equipment.^1,2^ The increasing complexity and cost of specialized instrumentation exacerbate this challenge, making it crucial to develop sustainable solutions that extend the usability of existing resources. The scarcity of equipment is especially relevant in research fields that require longer acquisition times. Upconversion (UC), the photophysical phenomenon exhibited by lanthanide ions, is one of such fields.^3,4^ These ions are characterized by their equally spaced 4f energy levels and extended excited-state lifetimes (microseconds to milliseconds), enabling the sequential absorption of multiple photons with identical near-infrared energy. A proper photophysical characterization requires a time-consuming power-dependent measurement of the steady state and time resolved emission spectra.^5^ The extended durations of these experiments can monopolize shared equipment, limiting access for other researchers. In addition, institutions with a financial constraint often choose to acquire conventional spectrofluorometers that are cheaper and have a broader user base, lacking the specific features needed for upconversion measurements.

The obsolescence of aging scientific instruments can pose an additional significant barrier, particularly when proprietary operating systems and software render them incompatible with modern computing platforms. Despite their inherent capabilities, high-quality instruments may be sidelined due to outdated software or operating systems, undocumented data formats, lack of technical support, or planned obsolescence. This results in unnecessary electronic waste and the loss of valuable research tools, which could otherwise be adapted for contemporary applications. Consequently, crucial investment in research infrastructure is wasted. There is a need for innovative solutions to reintroduce these legacy assets into the scientific ecosystem. Modernizing closed instruments to extend their useful life and capabilities with open technologies takes advantage of the existing mechanics, optics, and electronics while avoiding material and re-invention costs.^6^ This approach not only reduces financial and environmental burdens but also aligns with global sustainability efforts, particularly the United Nations Sustainable Development Goals (SDGs). Specifically, our refurbishment contributes to making advanced spectroscopy accessible for student training (Goal 4: Quality of Education), foster sustainable research infrastructure (Goal 9: Industry, Innovation and Infrastructure), lower barriers to participation in cutting-edge research (Goal 10: Reduced Inequalities), and promote equipment reuse over disposal (Goal 12: Responsible Consumption and Production).^7,8^

This issue of equipment obsolescence has contributed to a broader movement towards the development of affordable and accessible scientific instruments across various fields, including instrumentation, microscopy, spectroscopy, and data acquisition.^9,10^ Open hardware initiatives make designs and documentation freely available for anyone to use, build, and modify devices.^11,12^ For example, the Arduino platform has provided an inexpensive and easy-to-use electronics development platform based on a microcontroller (https://www.arduino.cc/). The OpenFlexure is an open-source microscope that costs less than 100 USD to build,^13^ and a Raspberry Pi-based spectrometer that costs less than 400 EUR has been recently developed.^14^ Open-source software and languages such as Python (http://www.python.org), which offer numerical and instrumentation libraries like NumPy^15^ and PyVISA,^16^ have played a pivotal role in lowering entry barriers and enabling rapid prototyping of data acquisition and analysis tools. It is worth mentioning that companies focusing on partial or full open-source hardware have emerged. For example, OpenBCI (https://www.openbci.com/) provides low-cost EEG systems for brain-computer interfaces, and Opentrons (https://www.opentrons.com/) offers liquid handling solutions for lab automation.

A fully open instrument is desirable but not always possible to achieve. By leveraging open-source hardware and software to refurbish high-quality scientific equipment, we can provide a cost-efficient solution to equipment scarcity and obsolescence. Here, we present a comprehensive refurbishment of the Horiba PTI QuantaMaster 400, an obsolete but research-grade spectrofluorometer. We replaced its outdated electronics, acquisition software, and operating system; and retrofitted to expand its functionality. The refurbished system can now perform upconversion steady-state and time-resolved spectroscopic measurements. We demonstrate the capabilities of our refurbished spectrofluorometer by characterizing well-known *β*-NaYF_4_/Yb^3+^, Er^3+^ upconversion nanoparticles (UCNPs). We measured their luminescence spectra as a function of incident power density at 976 nm and recorded lifetime decay curves, showcasing the instrument's enhanced functionality in studying nonlinear optical materials. This development has allowed our institution to start an upconversion research branch.

## 2 Experimental

### 2.1 Hardware

**Fig. 1:**
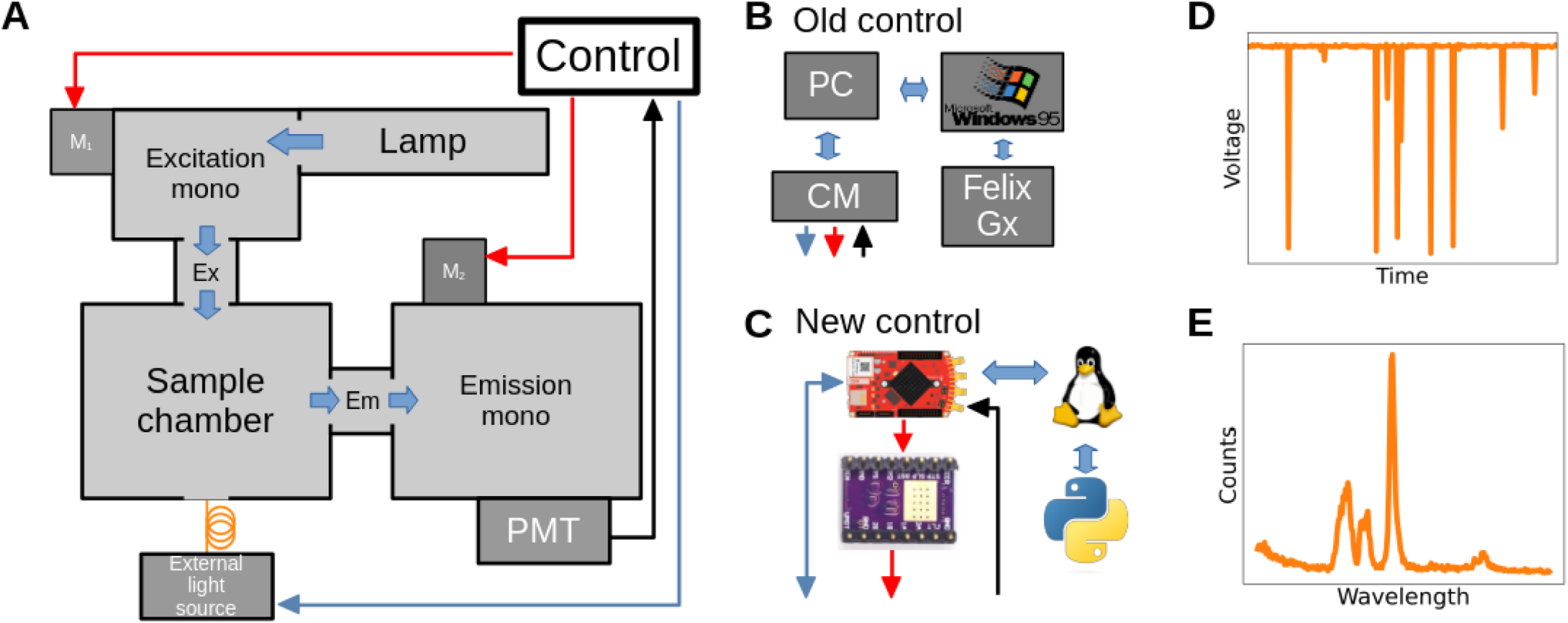
Schematic representation of the spectrofluorometer (A) diagram of the Horiba PTI QuantaMaster hardware. Red arrows represent motor and limit switch connectors, while black arrows represent BNC connectors. In the refurbished device, a 976 nm IR laser was added as an external light source. Blue: USB; Orange: fiber-optic. The path that light takes inside the spectrometer is represented in thick blue arrows. (B) and (C) Representation of the old and new instrumental control module respectively. (D) Representation of the raw signal measured by the PMT detector. (E) Spectrum of the sample constructed from the raw signals measured at each wavelength.

The Horiba QuantaMaster series comprises modular research-grade spectrofluorometers and multidimensional systems optimized for photoluminescence measurements. In this work, we refurbished a 30 year-old QuantaMaster 400 spectrofluorometer (diagram in Fig. 1A and picture in Fig. S1†). Wiring schematics for the system are not publicly available, necessitating a reverse engineering process prior to system upgrade. This system is equipped with a 75 W xenon lamp as the light source, providing a wide wavelength range from near-infrared (around 850 nm) to ultraviolet (around 300 nm). The sample chamber casing also allows for an external light source connected through a fiber optic cable, providing flexibility of the adapted setup to the experimental needs. The excitation and emission monochromators use gratings rotated by 200-step-*per*-revolution stepper motors (*M*_1_ and *M*_2_), each rated at 7 V and 0.7 A per winding, resulting in a wavelength selection resolution of 0.5 nm. The monochromator is equipped with electromechanical limit switches to verify if the wavelength boundaries have been reached.

**Fig. 2:**
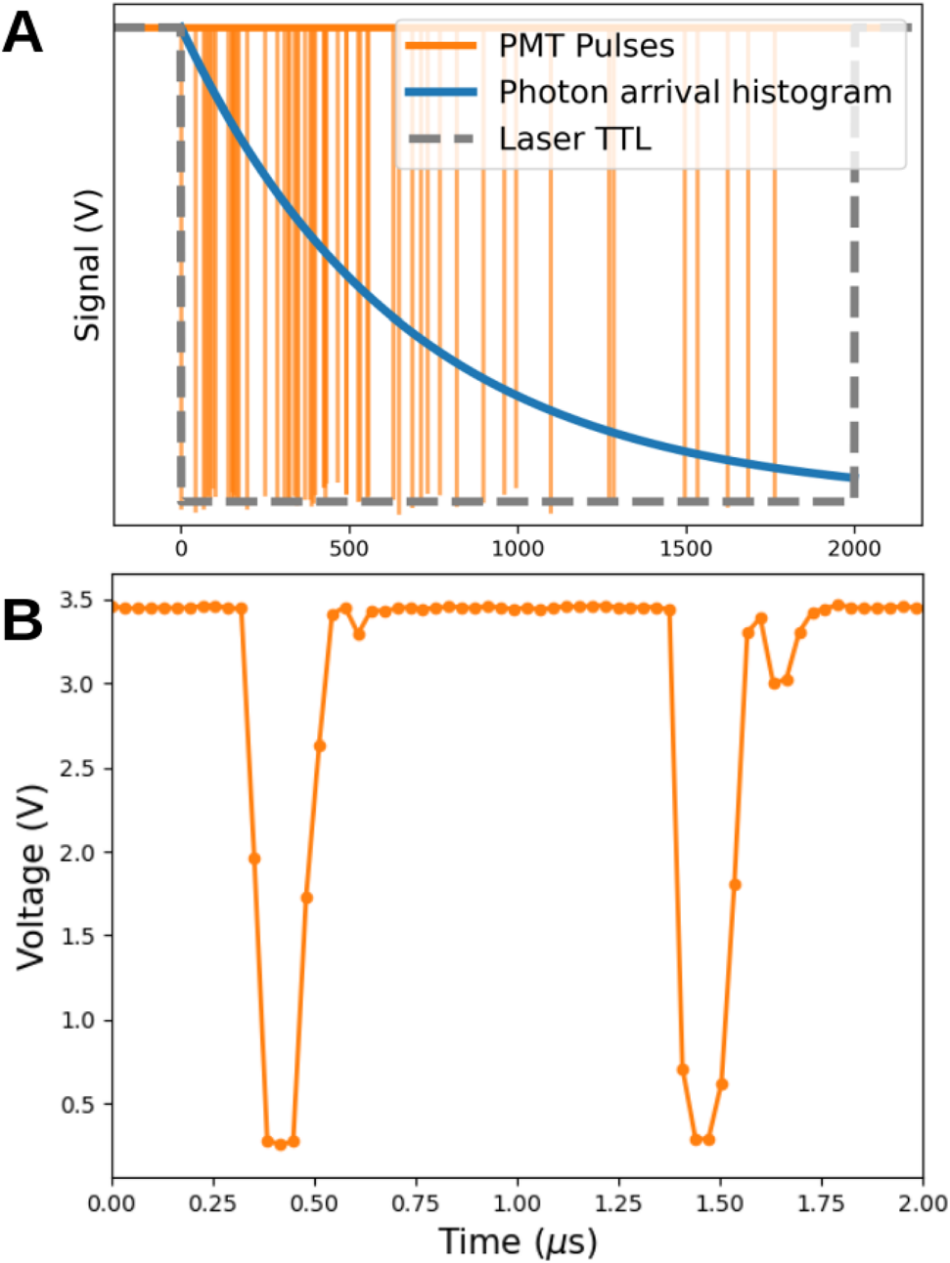
Lifetime measurement using a pulsed source. (A) Schematic view of a lifetime measurement. The TTL signal (gray) indicates whether the laser is on or off. The emission of the sample is detected by the PMT (orange), each photon is timed relative to the laser and the photon arrival time histogram (blue) is constructed. (B) The voltage signal of two pulses detected by the PMT and acquired by the Red Pitaya.

The detection system includes a PMT (PTI 810) connected to the control module (CM) through a BNC cable. The PMT is powered by an external high-voltage supply from the CM, polarizing the tube with 1000 V. Photons arriving at the PMT result in negative voltage pulses about 168 ns wide with a termination of −3.5 V into a 50 Ohm load (Fig. 1D and 2B). The CM connects *via* a flat ribbon cable to an outdated ISA interface board on a PC running Windows 95 and FelixGX, the proprietary acquisition and control software provided by Horiba. This software enables the user to acquire excitation and emission spectra (Fig. 1E) and control other system peripherals.

After evaluating all components, we decided to keep the high-quality industrial-grade optics, motorization, PMT, high-voltage power supply, and the overall casing. These components were found to be durable and fully functional. However, the control and detection electronics were bulky, closed-source, and obsolete. We replaced them with modern alternatives: a microcomputer with an integrated FPGA (Red Pitaya STEM LAB 125-14), which includes multiple analog and digital inputs/outputs, and two DRV8825 motor driver ICs for motor control (Fig. S2†). The FelixGX software was replaced with a custom Python-based program running on the Red Pitaya.

This modernization reduced the size of the control system significantly. The original motor driver of the CM (∼10 cm × 30 cm) was replaced with the compact DRV8825 motor driver ICs (∼2 cm × 3 cm), which are controlled *via* the Red Pitaya's digital I/O pins. The PMT pulses are now counted by software, which thresholds the acquired PMT voltage. To satisfy the Nyquist criterion for digitizing (168 ± 6) μs (FWHM) PMT pulses, the Red Pitaya's 14-bit analog input was set to a 32.25 MHz sampling rate. The maximum integration time per low-level call to the RP API is 8 ms due to it’s 2MB memory buffer. Longer times would require additional calls to the same API.

This upgrade not only replaced outdated technology but also laid the foundation for expansions. Specifically, it enabled us to retrofit the original Horiba PTI QuantaMaster 400 for UCNP research, particularly to study UCNP lifetimes in the millisecond range. Up-conversion is a highly nonlinear optical process, requiring precise control over excitation parameters such as average intensity, frequency, and duty cycle. In its original form, the spectrofluorometer was not equipped for dynamic up-conversion experiments, as it lacked both an IR light source and the necessary capabilities for time-resolved luminescence measurements. To address these limitations, we integrated an external modulatable IR light source into the system. Specifically, we mounted a BL976-SAG300 976 nm, 300 mW laser diode on a THORLABS ITC4020 thermoelectric controller (TEC) and laser diode driver unit. If the experiment does not need an intensity modulation or pulsed laser excitation, a simpler power source can replace the ITC4020. The laser diode was connected to the spectrofluorometer *via* a fiber optic cable routed through the external source input of the QuantaMaster 400. The ITC4020 allowed us to modulate the intensity, frequency, and duty cycle of the IR excitation, while providing a TTL signal for synchronization during lifetime measurements (Fig. 2). The 8 ms integration time acquisition window is adequate for most luminescent materials, but an incremental trigger delay can be employed for longer decay times. Measurement of rhodamine excitation and emission spectra with the original and the refurbished setups showed a perfect match not only in the position of the peaks but also in the shape of the curve (Fig. S3†). This demonstrates that the new motorization did not affect the wavelength calibration and the new counting electronics is equivalent to the original commercial setup. We characterize the reproducibility of stationary spectra UC measurements by repeatedly measuring the emission spectra of previously determined UCNPs.^17^ As before, the obtained wavelength and intensity profile matches and the previous results obtained using a commercial setup (Horiba Jobin Yvon Spectrofluorometer, Fluorolog 3) (Fig. S4†). To characterize the uncertainty of the lifetime response in time-dependent measurements, we first determined the jitter of the systems to be 2.4 μs using a reflective sample holder. This is two orders of magnitude smaller than the typical UCNP lifetimes. In addition, we measured the lifetime of previously characterized nanoparticles.^5^ Not only we observed a matching lifetime, but also the standard deviation of the average lifetime across 100 different experiments, with 10^5^ photons each is an order of magnitude smaller than the jitter.

The result is a platform capable for advanced UCNP optical characterization, built on the foundation of a refurbished and retrofitted legacy spectrofluorometer. Full details of the refurbishment process, including step-by-step instructions on how to build the spectrofluorometer, can be found in the ESI.†

### 2.2 Software

To control the new platform we developed a custom Python software that not only replaces the original proprietary FelixGX software but also significantly enhances the functionality of the spectrofluorometer by enabling user-defined acquisition protocols.

The core acquisition and control software runs on the microcomputer (Red Pitaya), which can be accessed remotely *via* a web interface from any computer, tablet or phone, replacing the original control PC. This provides flexibility for users to control the system without needing physical access to the spectrofluorometer. The codebase consists of three distinct layers (Fig. S7†) and includes reusable classes and functions, enabling users to develop custom acquisition scripts that suit their specific experimental needs. Users can modify the acquisition sequence, control additional external devices, or implement entirely new measurement protocols. This modularity facilitates future upgrades to the system and allows the software to adapt to different experimental setups or evolving research requirements. A straightforward graphical user interface (GUI) built using IPython's Jupyter Widgets is available, offering an intuitive platform for standard tasks like measuring both steady state and time-resolved emission and excitation spectra of fluorescent and phosphorescent samples (Fig. S8 and S9†). This user-friendly interface simplifies routine measurements while still allowing access to more advanced functionalities. For advanced users, the software's modular design is particularly advantageous. For example, we routinely use a measurement script that acquires an upconversion emission spectra, automatically locates the peaks and then acquires the time resolved emission.

A detailed step-by-step guide on installing the software, as well as instructions on performing measurements, can be found in ESI Sections B and C, respectively.

### 2.3 Synthesis of UCNPs

To demonstrate the capabilities of the refurbished spectrofluorometer, we synthesized *β*-NaYF_4_:Yb^3+^, Er^3+^ UCNPs with well known upconversion properties.

*β*-NaYF_4_:Yb^3+^(20%), Er^3+^(2%) nanoparticles were synthesized according to a reported procedure.^18^ YCl_3_·6H_2_O (0.843 mmol), YbCl_3_·6H_2_O (0.217 mmol), ErCl_3_·6H_2_O (0.026 mmol) were added to a 100 mL three-necked flask containing 6 mL of oleic acid (surfactant) and 15 mL of 1-octadecene (solvent). The solution was constantly stirred and heated to 150 °C under vacuum for 30 min for dissolution and then cooled down to room temperature. A solution containing 4.12 mmol of NH_4_F and 2.49 mmol of NaOH dissolved in 10 mL of methanol was added to the flask under stirring and heated slowly to 70 °C for 30 min until all the methanol was evaporated. Subsequently, the solution was heated to 300 °C for 2 h under argon and then cooled down to room temperature. The UCNPs were precipitated by adding ethanol, centrifuged and redissolved in hexane. This washing procedure was repeated three times. SEM images of the resulting nanoparticles can be seen in figure (Fig. S10†). The obtained particles have a rectangular prism shape, with square cross-sections measuring (47.9 ± 7.4) nm × (31.2 ± 3.9) nm. This geometry is favorable for photodynamic therapies, as it can diffuse through biological tissues, including blood vessels,^19^ multiphoton polymerization of 3d structures,^20^ and the construction of photonic devices.^21^

A small amount of the synthesized solid particles was deposited on a sample holder and placed into the fluorometer's sample chamber. We opted to measure them in solid state as powders and not to disperse them in a solvent to minimize the well known non-radiative mechanisms and to characterize them as such. The holder was fixed in place and remained stationary throughout all experiments, ensuring consistent illumination of the sample by the IR laser.

## 3 Results

### 3.1 Power dependent UCNPs dynamic spectrum

**Fig. 3:**
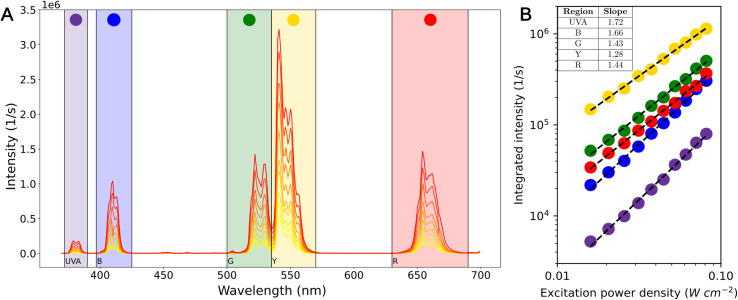
*β* − NaYF_4_:Yb^3+^, Er^3+^ UNCP power dependent spectrum. (A) Emission spectra under CW 976 nm excitation for different incident power densities, ranging from 16 mW cm^−2^ to 80 mW cm^−2^. From left to right, spectral regions labeled at the bottom of the figure named UVA, B, G, Y and R are highlighted. (B) Log–Log plot of the integrated intensity measurements for each of the spectral regions highlighted in (A). An inset table shows the slopes of a linear least squares fit of the data.

**Fig. 4:**
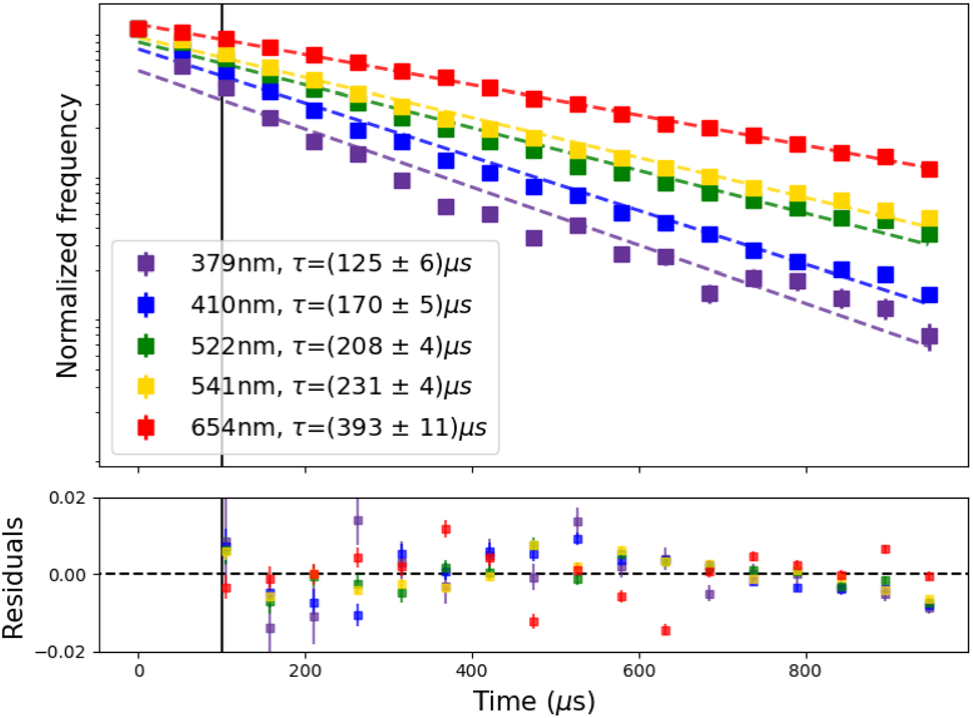
Photon time arrival histograms (squares) and exponential decay fits (solid lines, from 100 μs on) and its residuals. Lifetimes were measured at 976 nm, 0.087 mW cm^−2^ excitation power density.

This refurbished spectrometer, suitable for the optical characterization of UCNPs, allowed us to measure the power dependent dynamic spectrum of the synthesized UCNPs (Fig. 3 and 4) upon excitation with a 976 nm IR laser diode. Upconversion (UC) is a nonlinear optical process in which two or more photons are absorbed sequentially between equally spaced energy levels leading to the emission of light of shorter wavelength than the incident one. Given the particularly large lifetimes (μs to ms) and ladder-like energy levels of the rare earth ions, upconversion spectra of UCNP in the visible range can be observed even at low excitation densities. Such is the case for the emission spectra of UCNPs obtained at low power densities ranging from 16 mW cm^−2^ to 80 mW cm^−2^ (Fig. 3A). The static spectrum shows the well known emission peaks of Er^3+^ ions. The sharp peaks of Er^3+^ match the wavelengths reported in previous articles,^18^ which validates the wavelength accuracy of the refurbished monochromators. Starting on the lowest energy side, we labeled the emission regions as red (R, 630–690 nm), yellow (Y, 535–570 nm) and green (G, 500–535 nm) corresponding to transitions ^4^F_9/2_ → ^4^I_15/2_, and ^4^S_3/2_,^2^H_11/2_ → ^4^I_15/2_ respectively. The spectrum also shows blue (B, 397–425 nm) and ultraviolet (UVA, 372–390 nm) which arise from higher excited level transitions like ^2^H_9/2_ → ^4^I_15/2_ (410 nm) or ^4^G_11/2_ → ^4^I_15/2_ (383 nm).^3^ Upconversion emission intensity (*I*_UC_) is related to the excitation power density in a non-linear manner, *I*_UC_ = *P*^*α*^, where *α* is the effective number of photons involved in the absorption process per up-converted photon emitted, and *P* is the incident power.^22,23^ The *I*_UC_*vs. P* log–log plot (Fig. 3B) shows that for this power range each emission region is characterized by a given slope related with the number of photons involved in each process (Fig. 3B inset). These low values of the slopes can be interpreted in terms of the competition between the linear decay and the UC mechanisms responsible for the depletion of the intermediate excited states as documented in detail.^23^ If the linear decay dominates the depletion of the intermediate excited states the determination of the order of each excitation process are the number *n* of pump photons required to excite the emitting state. If upconversion becomes the main depletion process of the intermediate excited states, the slopes will depend on the UC mechanism (ETU or ESA), on the predominant decay route and the fraction of absorbed power. No change in these slopes were observed for each wavelength (UV, B, G, R) indicating that the photophysical mechanism stays the same. This is in line with the fact that cross relaxation and back energy transfer (BET) from Er^3+^ to Yb^3+^ are negligible at these low power limits.^24,25^

Furthermore, our expansion on the spectrofluorometer allowed us to measure the lifetime on the emission peaks of each of the spectral regions (Fig. 4). We fitted the photon arrival time histograms with a monoexponential decay curve, starting at 100 μs after excitation stopped to measure state de-population only. The measured lifetimes range from ∼125 to ∼400 μs, exhibiting a decrease at shorter wavelengths, corresponding to higher-energy excited states, which is consistent with an increased number of relaxation pathways. These values align well with previously reported lifetimes for *β*-NaYF_4_:Yb^3+^, Er^3+^ UCNPs,^26,27^ as summarized in Table S6 of the ESI.†

## Conclusions

Most proprietary scientific equipment is robust and reliable, but pose a limitation on its usability over time because of models being discontinued and the obsolescence of its control platforms and software. This particularly affects low-resource countries, where instruments with a wide range of applications are prioritized, which can result in specific research areas like upconversion underfunded. In this work, we refurbished a common Horiba PTI Quanta Master 400 spectrofluorometer, replacing its outdated control electronics, PC and software with an open-source alternative, while keeping its robust hardware components like the monochromator optics and PMT. Besides renewing the spectrofluorometer, we expanded its functionality to illuminate the sample with an external 976 nm IR laser with a tunable power supply to measure lifetimes in the millisecond range, and the ability to vary the optical power density. This hardware and software update allowed us to perform a complete optical characterization of UCNPs by measuring its power dependent emission spectrum and the lifetimes of each of its emission peaks, by executing a single python script with the measurement protocol.

This work highlights the transformative potential of open-source systems in revitalizing scientific instrumentation, extending their usability, and democratizing access to advanced research tools. It aligns with the United Nations Sustainable Development Goals (SDGs) by promoting sustainable research practices, reducing electronic waste, and expanding access to scientific education and innovation. By lowering barriers to participation, it demonstrates how open science and sustainability can drive inclusive scientific progress. By enabling precise and adaptable measurements at a fraction of the cost of proprietary systems, this approach paves the way for more inclusive scientific exploration, particularly in resource-limited settings, fostering innovation and collaboration across diverse research fields.

## Data availability

The data analysis scripts of this article are available in the interactive notebook https://www.github.com/tdinapoli/refurbishedPTI.

## Author contributions

Tomás Di Napoli: Software, methodology, investigation, writing – original draft, formal analysis. Juan M. Bujjamer: Conceptualization, investigation, methodology, writing – reviewing and editing. Marcos Illescas: Investigation, validation. Beatriz Barja: Supervision, funding acquisition, writing – reviewing and editing. Hernán E. Grecco: Supervision, conceptualization, project administration, funding acquisition, formal analysis, writing – reviewing and editing.

## Conflicts of interest

There are no conflicts to declare.

## Supplementary Material

NA-OLF-D5NA00330J-s001
