# Supplementary material for: Giving new life to an outdated spectrofluorometer for static and time-resolved UCNP optical characterization
Source: Nanoscale Adv. 2025 May 27;7(13):4214–20. doi: 10.1039/d5na00330j (PMC12139447; doi:10.1039/d5na00330j)
Supplement: NA-007-D5NA00330J-s001 [file NA-007-D5NA00330J-s001.pdf]

Supplementary Information (SI) for Nanoscale Advances.
This journal is © The Royal Society of Chemistry 2025

Article title

Giving new life to an outdated spectrofluorometer for static and time-resolved UCNP optical characterization

Authors

*Tomás Di Nápoli (1), Juan M. Bujjamer (1, 2), Marcos Illescas (3, 4), Beatriz Barja (3, 4), Hernán E. Grecco (1, 2, *)*

Affiliations

1. Universidad de Buenos Aires, Facultad de Ciencias Exactas y Naturales, Departamento de Física. Buenos Aires, Argentina.
2. CONICET - Universidad de Buenos Aires, Instituto de Física de Buenos Aires (IFIBA). Buenos Aires, Argentina.
3. Universidad de Buenos Aires, Facultad de Ciencias Exactas y Naturales, Departamento de Química Inorgánica, Analítica y Química Física. Buenos Aires, Argentina.
4. CONICET - Universidad de Buenos Aires, Instituto de Química Física de los Materiales, Medio Ambiente y Energía (INQUIMAE). Buenos Aires, Argentina.

A. Refurbished Horiba PTI QuantaMaster 400 build instructions

Required materials for the build process can bee seen in table (S1).

Component	Number	Cost per unit (US$)	Total cost (US$)	Needed for refurbishment	Source
DRV8825 stepper motor driver	2	15.95	31.9	yes	Link
Red Pitaya STEMlab 125-14	1	426.8	426.8	yes	Link
ITC4020 benchtop laser diode controller	1	5114	5114	no	Link
BL976-SAG300 laser diode	1	727	727	no	Link

Table S1: The parts needed for refurbishment are the ones needed to reproduce the original instrument's funcrionality. The ones not needed for refurbishment are used for the instrument's feature enhancement as measuring lifetimes. Both the refurbishment and upgrade can be made with different parts that have similar characteristics as the ones listed here.

Refurbished Horiba PTI spectrometer build process can be organized in five steps have to be taken: (1) connect excitation motor M_1 (**Fig. S1**), (2) connect excitation limit switch, (3) connect emission motor M_2, (4) connect emission limit switch, and (5) connect the PMT output. Throughout the build process every power source should be off until everything is connected properly as in schematic (**Fig. S2**). Make sure the GND voltage is the same for every connection. **Warning:** make sure the driver current limit is properly configured, and the pins are correctly connected, or else there is a risk of running too much current over the motors' windings and breaking them.

1. **Connect excitation motor**

   (a) Connect pins P6 and P7 of the RP to the STEP and DIR pins of the motor driver, respectively (**Fig. S2**).

   (b) Connect the remaining pins: taking 3.3 V from the RP as digital high, pull the motor driver $\overline{\text{SLEEP}}$ and $\overline{\text{RESET}}$ pins high, and connect the logical GND to the RP ground. The remaining pins $\overline{\text{ENABLE}}$, M0, M1, M2 and $\overline{\text{FAULT}}$ can be left floating.

   (c) Adjust the current output limit of the DRV8825 to the maximum supported for your stepper motor, which in our case being motor M061CS02 is 0.7 A.

   (d) Provide the motor power supply by connecting pins VMOT and GND to a 12 V power supply that can provide at least 2 × *current limit* of the motors. Connect a 100 μF decoupling capacitor in parallel.

   (e) Connect the motor driver to the stepper motor: disconnect the original M_1 motor connector and place driver pins A1 and A2 of the DRV8825 to pins 1 and 7 of the stepper motor, and driver pins B1 and B2 to pins 3 and 5 (**Fig. S1B**).

2. **Connect excitation limit switch**

   (a) Provide 5 V and GND to pins 1 and 2 respectively of the limit switch next to the motor M_1 (**Fig. S1C**).

   (b) Attach pin 3 of the limit switch to digital pin P2 of the RP for M_1 limit switch with an external pull up resistor to logical 3.3 V of the RP.

3. **Connect emission motor**
   (a) Connect pins P4 and P5 of the RP to the STEP and DIR pins of the motor driver, respectively.
   (b) Repeat steps (b) through (e) of item (1) but for motor M_2.
4. **Connect emission limit switch**
   (a) Provide 5 V and GND to pins 1 and 2 respectively of the limit switch next to the motor M_1 (**Fig. S1C**).
   (b) Connect pin 3 of the limit switch to digital pin P3 of the RP for M_2 limit switch with an external pull up resistor to logical 3.3 V of the RP.
5. **Connect the PMT output**
   (a) Connect the BNC-SMA cable to RP's Analog 1 and set high voltage mode.
   (b) Connect a BNC T connector to the BNC end of the BNC-SMA cable connected to RP's Analog 1.
   (c) Connect the 50 Ohm termination to one of the BNC T connector ends for a pulse conformation of 40 to 100 ns.
   (d) Connect the other end of the T connector to the output of the PMT with a BNC-BNC cable.

After these steps, the instrument is ready to perform stationary measurements.

To add the functionality of measuring lifetimes with the Horiba PTI spectrometer, the previous instructions, the following additional building steps have to be performed:

1. Connect the external light source to the external port (**Fig. 1**) through a fiber optic cable.
2. Connect the ITC4020 USB B port to the RP's USB A port.
3. Connect the R3 BNC output (ITC4020's 5 V TTL trigger output) on the rear panel of the ITC4020 to a BNC to SMA adapter, and then connect it to analog input 2 on the RP and configure that channel with high voltage mode.

Our software is designed to work with ITC4020 laser controller, but a different one can be used if control classes are added to the software.

B. Software

A Python software that both succeeds in replacing the original software FelixGX and enhances the spectrometer functionalities by enabling the user to program specific acquisition protocols was developed. It is highly modular and it is built in three distinct layers (**Fig. S7**)

- **Graphical user interface (GUI)**: provides the same acquisition tools as the FelixGX software. Web based, uses IPython's Jupyter Widgets.
- **Class based Spectrometer**: orchestrates the hardware for specific measurement protocols (e.g. acquire an emission spectra). Easy to use from a Python script or command line. It is used by the GUI.
- **Class based devices**: controls individual parts of the spectrometer such as the photon counter, the pulse laser and the monochromator, allowing to generate custom measurement protocols. Lower level classes to control the motor or the Red Pitaya are also available.

Follow the instructions in this URL to install our Python package in your RP. Once the hardware components are connected, a calibration of the monochromator motors and the Red Pitaya's analog inputs has to be performed. The Red Pitaya's analog inputs calibration steps are explained in its documentation.[1] For the calibration of the monochromators we developed a command line interpreter to do a step by step calibration that generates a yaml file with the calibration parameters. The yaml file consists of the parameters described in **Table S2**.

Parameter Name	Data Type	Description
`greater_wl_cw`	bool	True if wavelength increases clockwise, False if counterclockwise.
`max_wl`	float	Maximum wavelength (in nanometers) allowed by the Monochromator API.
`min_wl`	float	Minimum wavelength (in nanometers) allowed by the Monochromator API.
`wl_step_ratio`	float	Wavelength change (in nanometers) per step of the stepper motor.
`home_wavelength`	float	Home wavelength (in nanometers).

Table S2: **Monochromator API Parameters**.

In order to run the command line interpreter and to generate this file for the emission monochromator, you have to open a Python terminal and execute the following code:

```python
from refurbishedPTI import Spectrometer
spec = Spectrometer.constructor_default()
spec.emission_mono.calibrate()
```

This will open a command line menu that guides the user for the generation of the configuration file. When the calibration process is done, the command `save_to_yaml` saves the configuration file to the RP's directory path returned by the method `.get_config_path()` of the Motor class. Once the emission monochromator calibration file is done, you can proceed to output the excitation monochromator calibration file in the same way by executing

```python
spec.excitation_mono.calibrate()
```

and repeat the same step followed for the emission monochromator. 1

C. Operation instructions

There are two ways of operating the refurbished Horiba PTI spectrometer: via the Python API for the hardware, and via the IPython Widgets Jupyter Notebook GUI. For both modes of operation, every instrument listed in the explanation of section A has to be connected, and the PMT and the lamp have to be powered on. Adjust the Em and Ex slits (**Fig. 1**) according to the experiment needs. Once these steps are completed, proceed with the following sections for script or GUI operation mode.

C.1 GUI operation mode

The spectrometer's GUI is contained in a Jupyter Notebook that allows the user to change the instrument's parameters, displaying Jupyter Widgets. The GUI is composed of two sections: the parameters panel and the graphs panel (**Figs. S8** and **S9**). The parameters panel consists of dropdown menus, buttons, and text fields to specify the measurement and measurement file parameters. The graphs panel consists of two graphs, one for the

[1] https://redpitaya.readthedocs.io/en/latest/appsFeatures/systemtool/calibration.html

spectrum measurements and another for the lifetime measurements. To initialize the GUI operation mode of the refurbished spectrometer, open the gui.ipynb notebook located at `/home/jupyter/refurbishedPTI/gui.ipynb`. Run the first notebook cell with the code

```
from refurbishedPTI.gui import Gui
gui = Gui()
```

and follow instructions in the following paragraph to take a spectrum or a lifetime measurement (**Figs. S8** and **S9**).

Take a spectrum or lifetime measurement by selecting options **Spectrum** or **Lifetime** on the **Measurement type** dropdown. Use the GUI components (**Tables S3** and **S4** to specify the measurement parameters and start acquiring. Once the measurements finish, save and manipulate the data with the GUI components (**Table S5**).

**Parameter Name**	**Description**
Spectrum type	**Emission**: fixed excitation monochromator and scanning emission monochromator.
	**Excitation**: fixed emission monochromator and scanning excitation monochromator.
	**Laser**: scanning emission monochromator external laser excitation.
Static monochromator wavelength	Fixed monochromator wavelength (nm).
Starting wavelength	Starting wavelength of the scanned wavelength range (nm).
Ending wavelength	Ending wavelength of the scanned wavelength range (nm).
Wavelength step	Difference in wavelength between each data point (nm).
Acquire	Starts the measurement.

Table S3: **Spectrum measurement configuration parameters**.

**Parameter Name**	**Description**
Pump power	Laser pump power (mW).
Frequency	Laser on and off TTL signal frequency.
Duty Cycle	Duty cycle of TTL signal in %.
Emission monochromator wavelength	Wavelength at which the lifetime will be measured (nm).
Amount of counts	Amount of counts that will be measured until the measurement ends.
Starting time	Time after trigger before start counting (ms).
Ending time	Time after trigger before stop counting (ms).
Acquire	Starts the measurement.

Table S4: **Lifetime measurement configuration parameters**.

C.2 Python script operation mode

C.2.1 Taking a spectrum measurement

The software we developed allows the user to take both emission and excitation spectrum measurements. For both cases, the first step is to initialize the spectrometer

```
from refurbishedPTI.instruments import Spectrometer
spec = Spectrometer.constructor_default()
```

The `constructor_default()` method initializes the spectrometer by passing to it the emission monochromator and excitation monochromator initialized by the class Monochromator in the instruments.py module,

Parameter Name	Description
Measurement filename	Select a filename and directory where the measurement will be saved once the **Save** button is pressed. If no filename is selected at the time of pressing the **Acquire** button, the filename will be the current date and time.
Selected measurement	Select a measurement to **Save** it or **Delete** it.
Save	Save measurement with selected filename, directory, and format.
Delete	Delete selected measurement.
Save to	File format for the saved measurement. Options:
	**pickle**
	**csv**
	**excel**

Table S5: **Spectrum measurement file configuration parameters**

.

and the redpipy Oscilloscope object (redpipy is a Python package that is a more pythonic wrapper over the RP'sobject Python API). Both Monochromator instances are initialized with the `emission_init.yaml` and `excitation_init.yaml` configuration files respectively saved in the directory path returned by the Monochromators' method `.get_config_path()`. These files specify the RP's pins used for each motor driver pin and the monochromator calibration file path. Alternatively, the user can initialize different instances of excitation and emission monochromators and oscilloscope and pass them to the Spectrometer class to initialize it.

After the Spectrometer is initialized, both monochromators have to be homed, that is, they have to be moved to the wavelength that triggers the limit switch output. That can be done with the command

```
spec.home()
```

Alternatively, the current wavelength of each monochromator can be set by

```
spec.excitation_mono.set_wavelength(ex_wl)
spec.emission_mono.set_wavelength(em_wl)
```

where `em_wl` and `ex_wl` are variables with the value of the current wavelength of each monochromator. Finally, to get the emission spectrum do

```
df = spec.get_emission(
    integration_time=0.01,      # In seconds
    excitation_wavelength=400, # In nanometers
    starting_wavelength=500,   # In nanometers
    ending_wavelength=700,     # In nanometers
    wavelength_step=1          # In nanometers
)
```

where `integration_time` is the integration time of each data point, `excitation_wavelength` is the wavelength at which the lamp monochromator will be set during the measurement, `starting_wavelength`, `ending_wavelength` and `wavelength_step` are the starting and ending wavelengths of the spectrum, and the wavelength between each data point respectively. This method returns a Pandas DataFrame with columns wavelength, photon counts per second and integration time. The DataFrame also has `attrs` values that state the type of the spectrum (emission, excitation, or laser) and what wavelength the static monochromator was at during the experiment. These are optional arguments, for which the default is the minimum and maximum wavelength, and the minimum wavelength step. The `get_emission` method has another relevant optional argument called `feed`. The `feed` argument is a callable that is executed between each measurement, with a dictionary with keys `wavelength`, `counts` and `integration_time` as its argument, representing the measurement of one data point of the spectrum.

The process to get an excitation spectrum is analogous but the method `get_excitation` has to be used,

whose arguments are the same as `get_emission` except for `excitation_wavelength` which is replaced by `emission_wavelength`.

C.2.2 Taking a lifetime measurement

To perform a lifetime measurement, both the laser diode power source and the spectrum have to be configured. To control the laser diode the ITC4020 controller has to be initialized.

```
from refurbishedPTI.instruments import ITC4020
itc = ITC4020()
```

With the laser controlled initialized, its parameters can be set via the ITC API. These parameters are a subset of the parameters found on the instrument's user manual. In this example, only the parameters relevant to the lifetime experiment will be set, the duty cycle, the laser current and the frequency. Before setting those values, the ITC has to be in Quasi-continuous-wave (QCW) mode, where the pump power is switched on for short time. Performing the previously mentioned commands results in the following code.

```
itc.qcw_mode = True
itc.frequency(100) # Sets frequency at 100 Hz
itc.current(0.1)   # Sets current at 0.1 A
itc.duty_cycle(80) # Sets the duty cycle at 80%
```

Once these parameters are set, the laser can be turned on with

```
itc.laser_on(True)
```

Finally, with the Spectrometer object initialized as described in section C.2.1, configure it and acquire the arrival time of the photons with

```
spec.set_decay_configuration()
counts = spec.acquire_decay(
    t0=0,                 # In ms after trigger
    tf=2,                 # In ms after trigger
    amount_counts=1000, # Amount of counts
)
```

where `t0` is the time between the trigger and the beginning of counting, `tf` is the time between the trigger and the end of counting, and `amount_counts` is the amount of counts until acquiring stops. This method returns a Pandas DataFrame with a column `arrival_times` with the different photon arrival times.

D. Refurbished spectrometer functionality validation

To validate that the new counting electronics were not biasing the results, we measured the excitation and emission spectrum of a rhodamine 6B solution with both the original hardware and the refurbished spectrofluorometer. Figure (S3) shows the comparison between all four spectrums, showcasing the same intensity ratio between the emission peaks. Furthermore, we measured the stationary spectrum of previously reported gadolinium(III) oxide upconversion nanoparticles (UCNPs) doped with 5:1 Er and Yb lanthanides[1] multiple times to account for reproducibility (**Fig.** S4). We compared the legacy counting and control hardware and software with the refurbished one, always using the added external IR laser for excitation.

The RP's timebase of 31.125 MHz, corresponding to 30 ns between samples, allowed us to characterize the delay and jitter of the laser after the RP's oscilloscope is triggered, resulting in $(1.9 \pm 1.2)\mu s$. The estimation of the delay and jitter of the laser was constructed by recording the arrival time of the first photon detected at 980 nm after the TTL (see sect. 2.1) signaled on. To do this, the laser beam was focused on an empty sample

holder to reflect the incoming light to the emission monochromator, reaching the PMT afterwards. The delay corresponds to the mean of the arrival times, and the jitter to the standard deviation. This lets us define the instrumental error on the lifetimes as the laser jitter $\pm 2.4\mu$s, comparable to the uncertainty reported in recent work in the field[2]. This error is two orders of magnitude lower than the typical lifetimes of UCNP. To validate the lifetime measurement features we added to the spectrometer, we measured the lifetime of upconversion nanoparticles by exciting the sample at 980 nm and setting the emission monochromator at 410 nm. We then fitted the photon arrival times with a monoexponential fit model $f(t) = ae^{-t/\tau}$ where a and τ are the fitted parameters, and τ corresponds to the sample lifetime. The result of the arrival times and the fit are shown in figure (S5) resulting in $\tau_1 = 167\mu s$, which is comparable to previously published results[3].

To account for reproducibility in lifetime measurements, we measured the lifetime of previously reported UC-NPs[1] at 655 nm emission multiple times for different amounts of total measured counts (**Fig. S6**). We show that the different measurements show the same trend by repeating the experiment 100 times for different total amounts of photons detected, ranging from 100 photons to 10^5 photons. We also compare the mean photon arrival time for each experiment.

We have tested these modifications both for our setup and for a similar model released also in the 1990's and another one released in the 2010's. Testing on a completely different hardware setup remains to be done, yet we believe that this refurbishment — perhaps with minor modifications — can be applied to any spectrometer with monochromators driven by stepper motors, and a PMT for light detection.

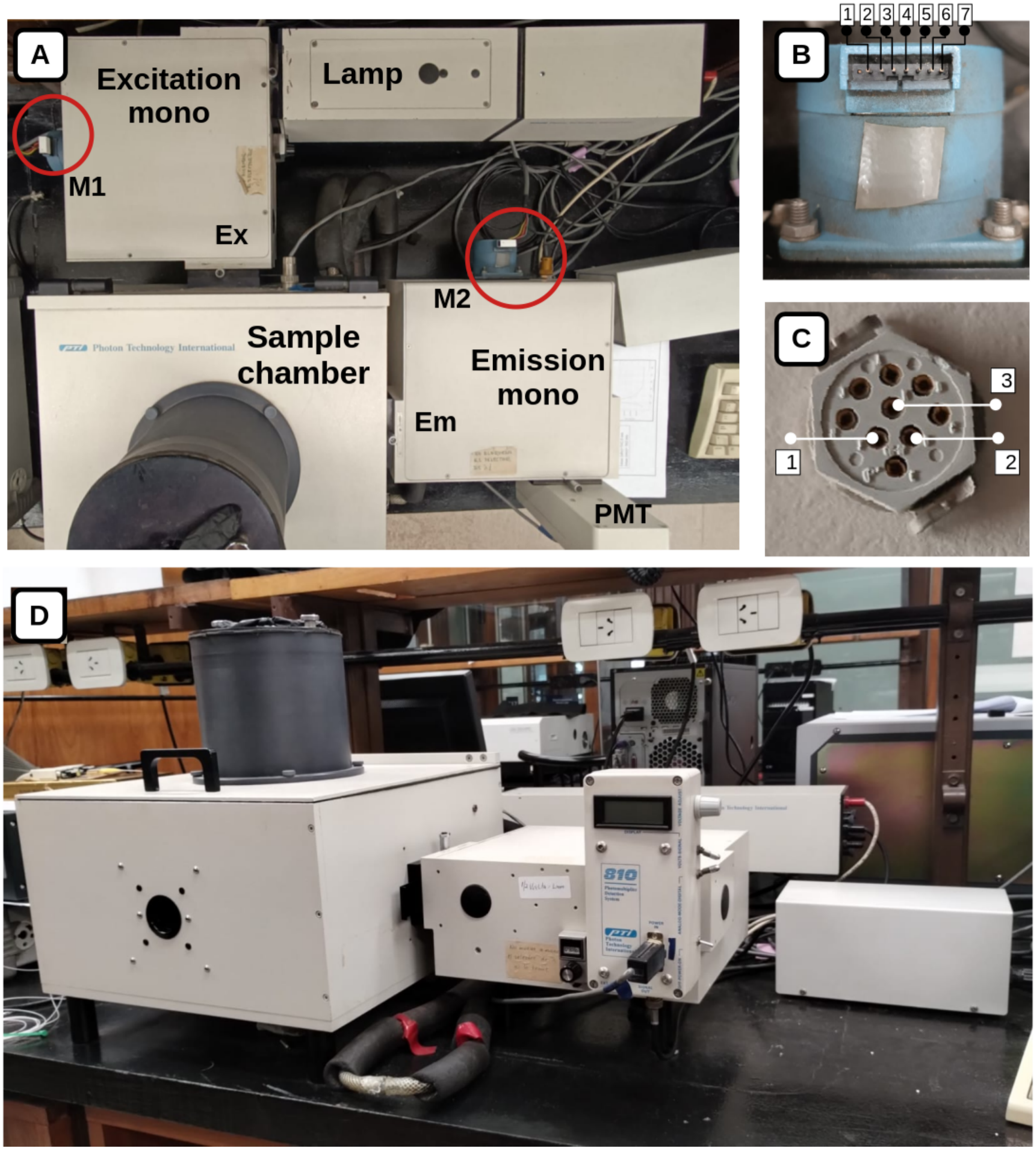

Figure S1: **Horiba PTI QuantaMaster 400 picture**. (**A**) Picture of the whole spectrometer. Circled in red the monochromators' motors and limit switches. (**B**) Stepper motors pin diagram. The only used pins for the refurbished version are 1 and 7, and 3 and 5, which correspond to each motor winding respectively. (**C**) Limit switches pin diagram. (**D**) Full image of the spectrofluorometer.

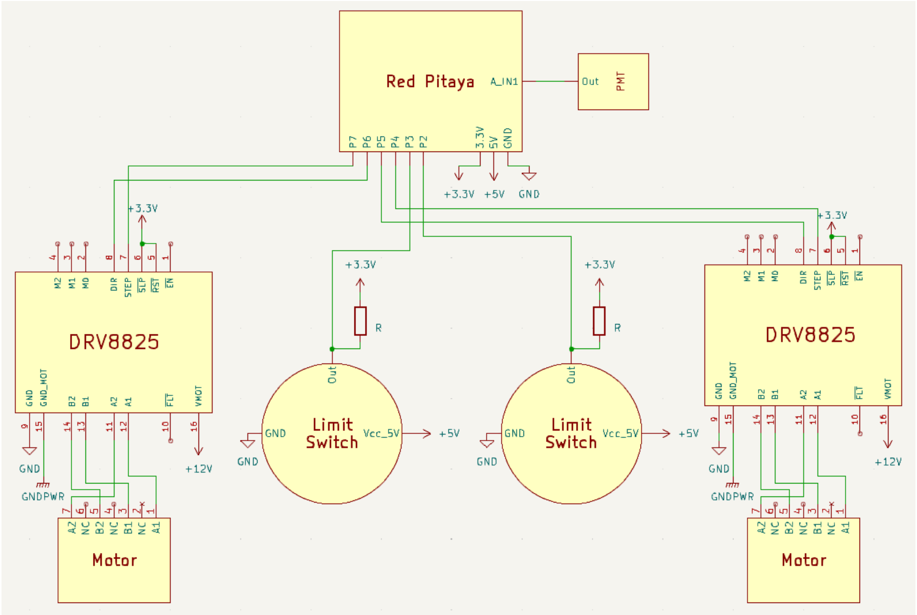

Figure S2: **Connection diagram**.

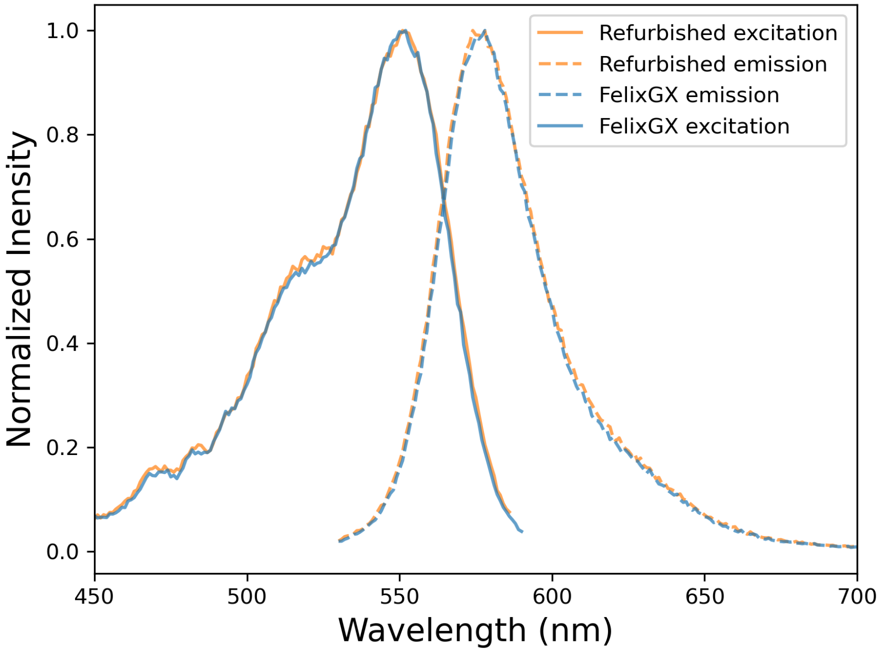

Figure S3: **Experimental validation of the spectral measurement**. Excitation (solid) and emission (dashed) spectrum of rhodamine 6B measured with original Horiba PTI QuantaMaster400 hardware and software (blue) and the refurbished (orange) version.

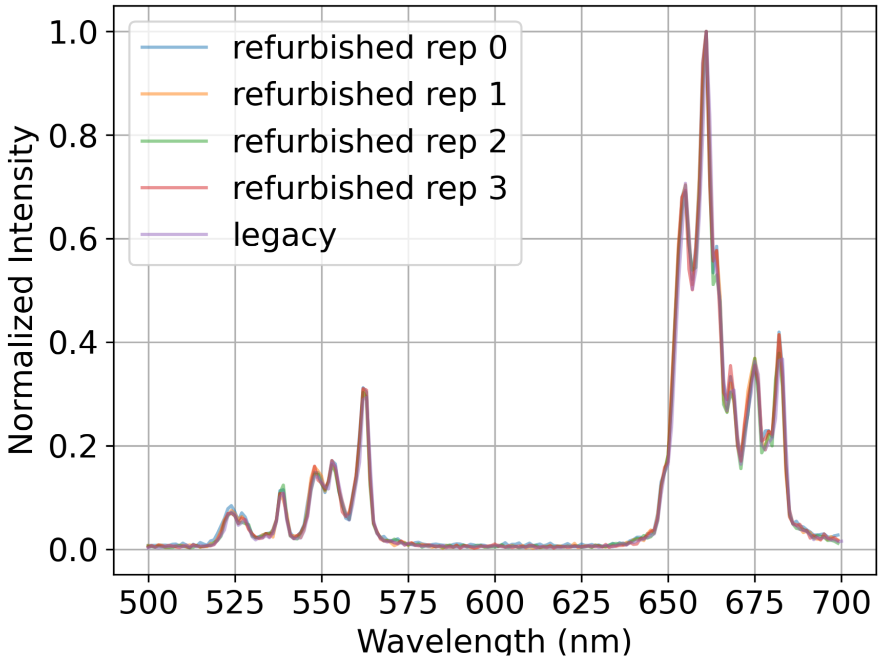

Figure S4: **Validation of stationary spectrum measurement reproducibility**. Emission spectrum of previously reported UCNPs[1]. In light blue the spectrum measured with the legacy control hardware and software. All other curves represent the spectrum measured 4 different times with the refurbished instrument.

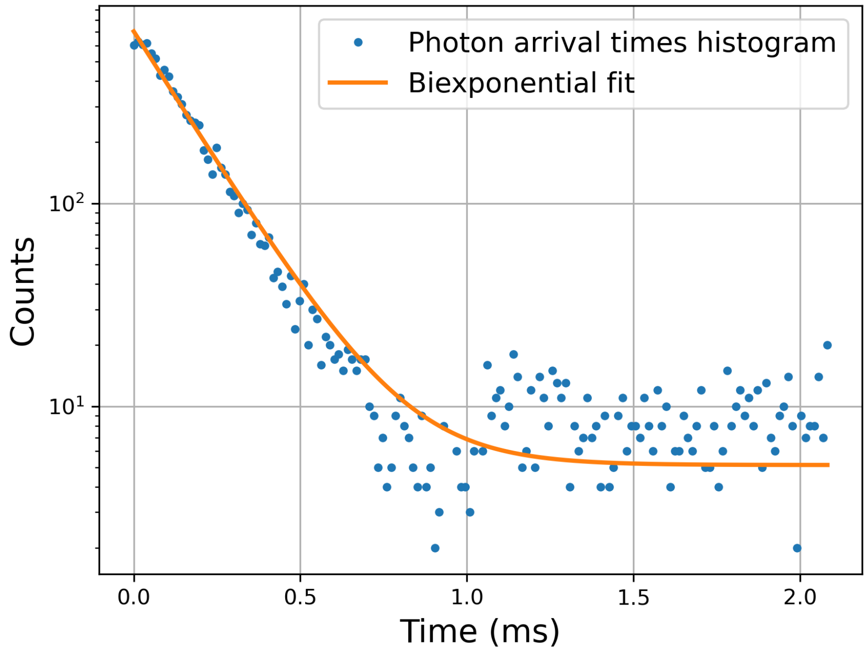

Figure S5: **Experimental validation of the lifetime measurement** Emission of UCNPs at 410 nm excited at 980 nm. In blue, the photon's arrival time histogram. In orange, the biexponential fit to the distribution.

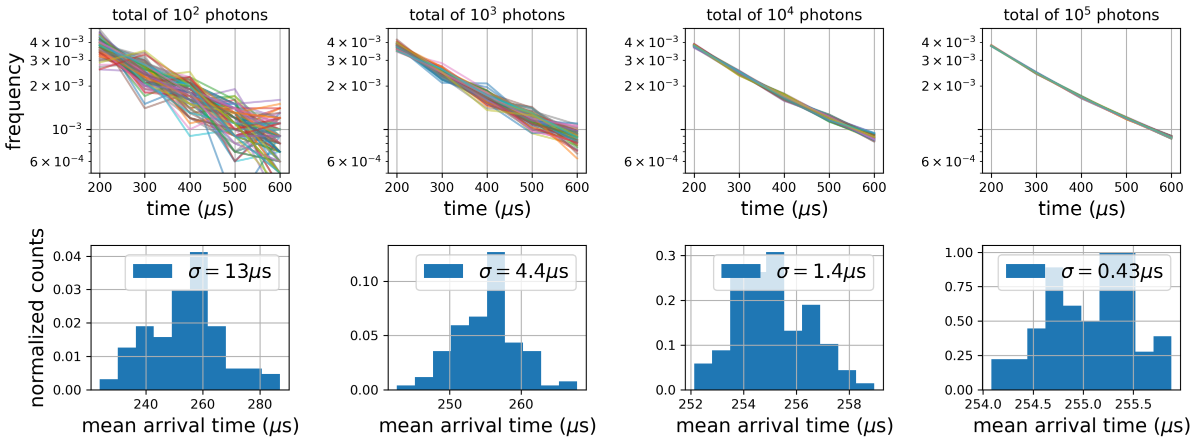

Figure S6: **Validation of lifetime measurements reproducibility.** Emission of previously reported UCNPs[1] at 655 nm, excited at 980 nm. Each column represents a set of 100 different lifetime measurements with different total amounts of counted photons. On the top row, the arrival time of the photons normalized. On the bottom row, the mean arrival time histogram for each experiment.

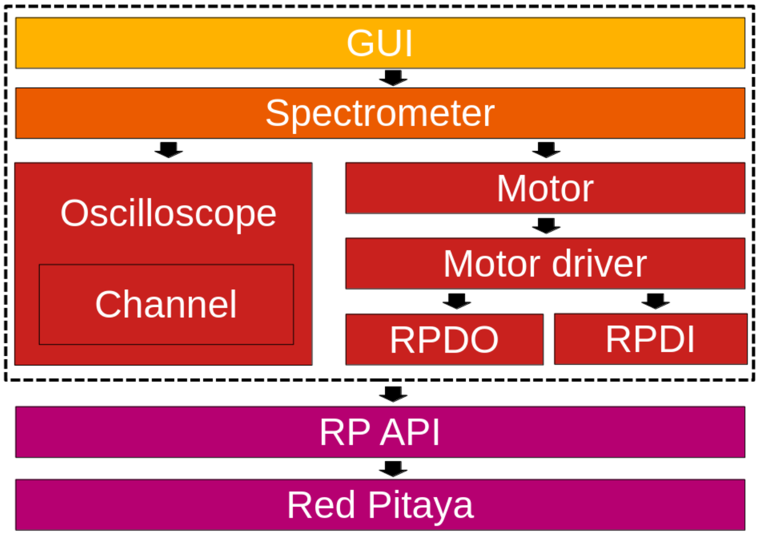

Figure S7: **Structure of the software**. Each element of the software is ordered from high level (**top**) to low level (**bottom**). Inside the dashed line black box In yellow, the two ways the end user can interact with the software. In orange, the refurbished instrument API classes. In red, the RP's hardware API.

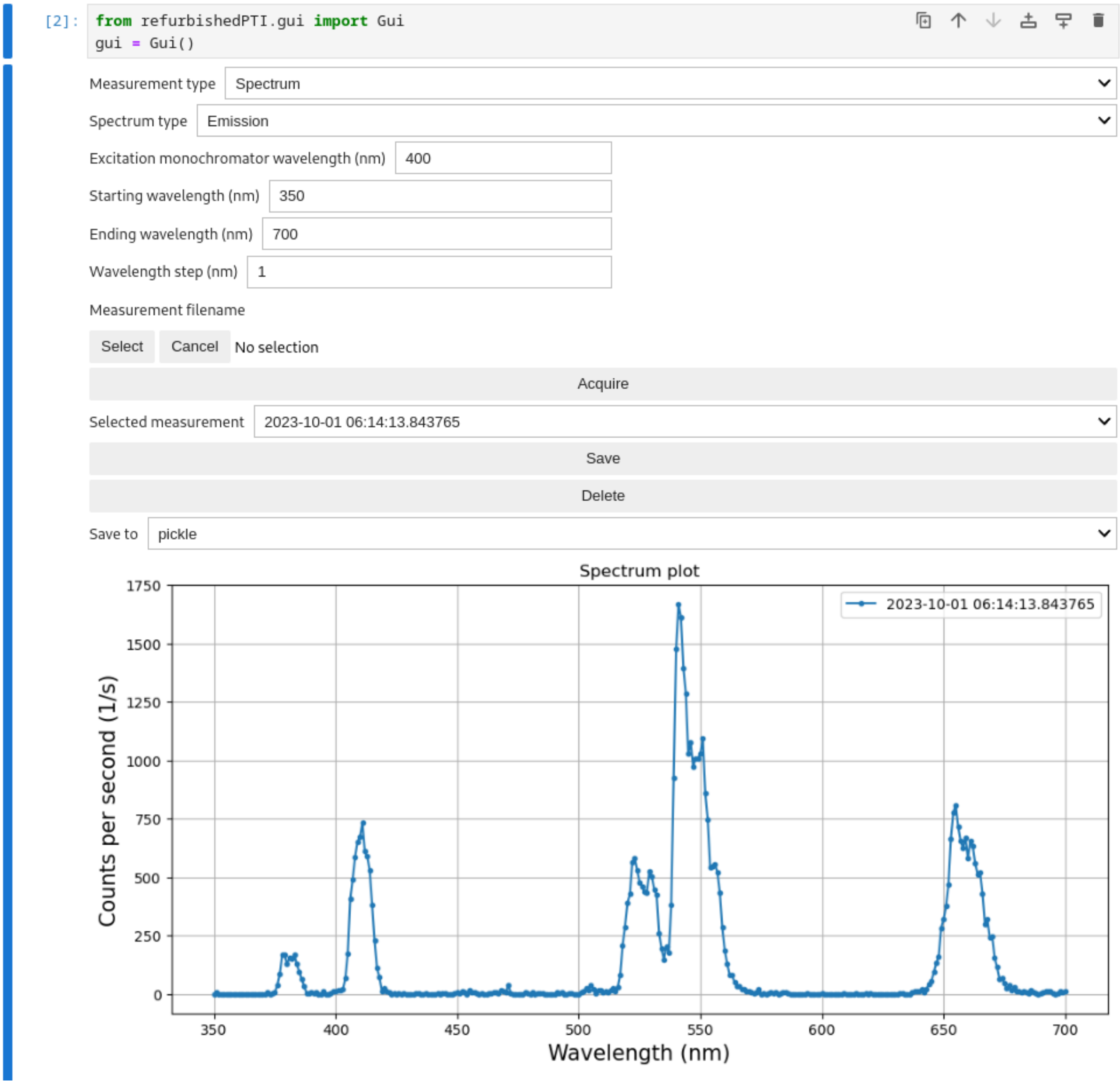

Figure S8: **Open source spectrum measurement GUI.**

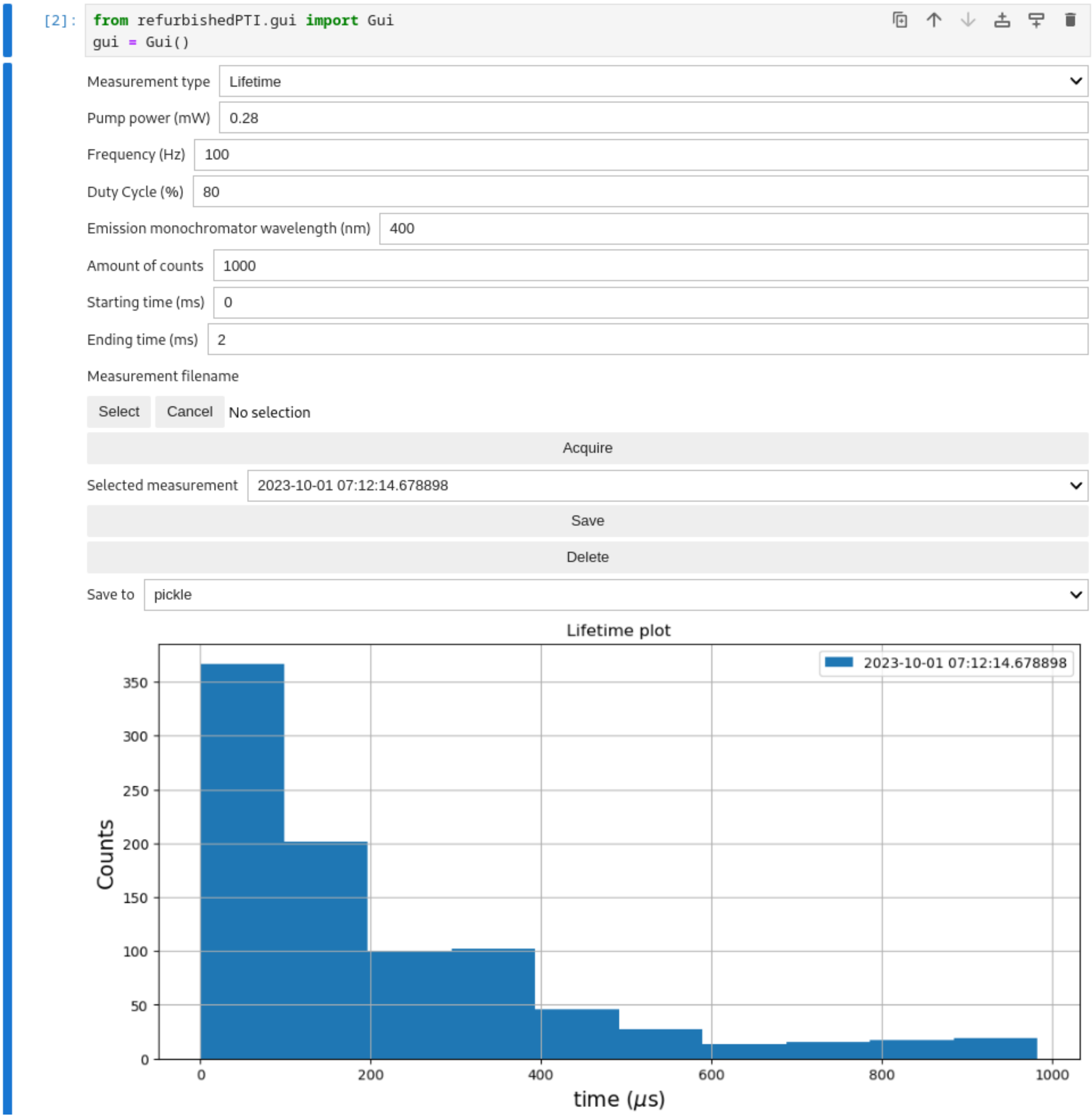

Figure S9: **Open source lifetime measurement GUI**.

E. β-$NaYF_4$:Yb^{3+}, Er^{3+} UCNPs SEM images

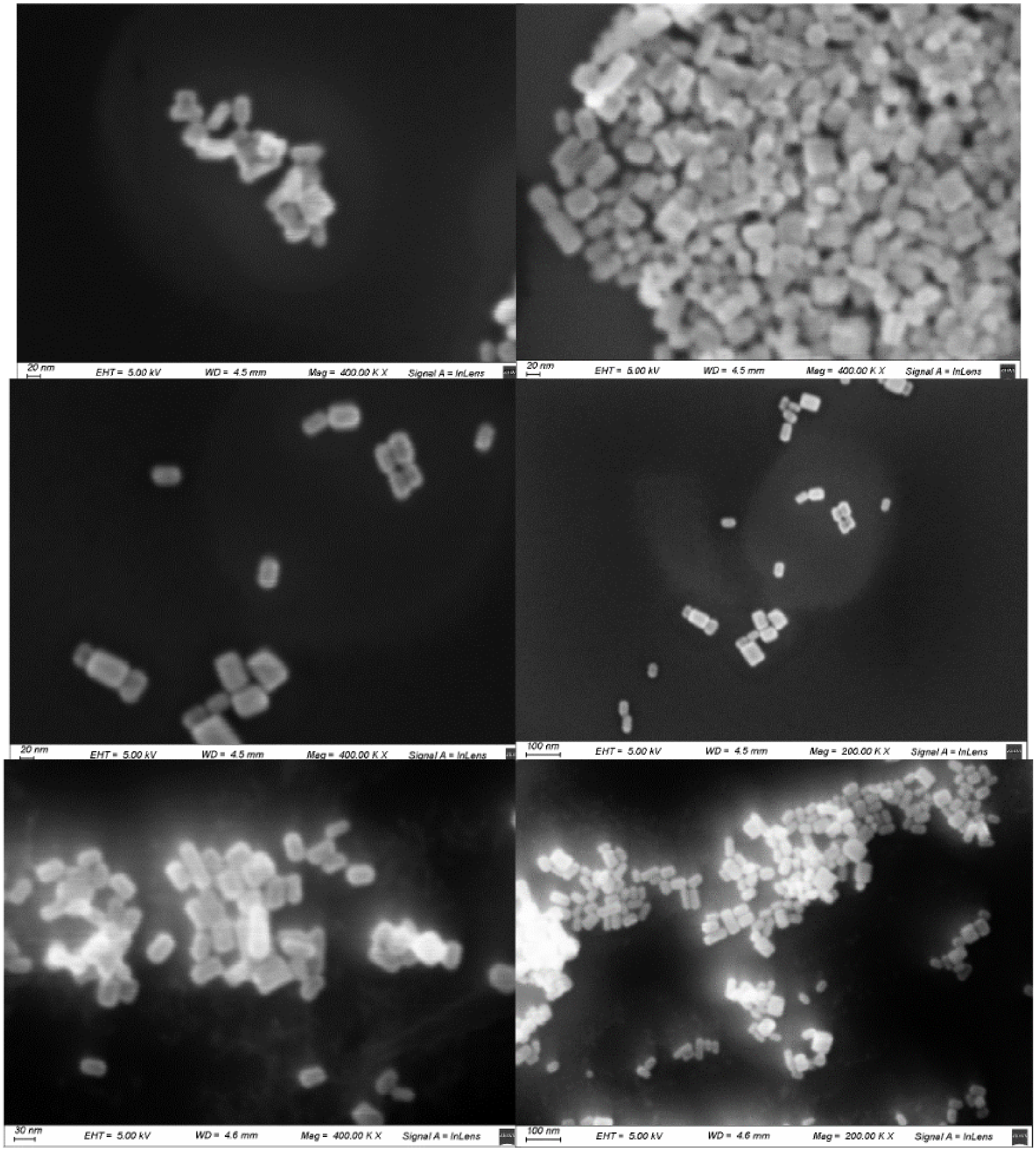

Figure S10: SEM images of the synthesized nanoparticles.

	Size (nm)	Emission Wavelength (λ_{em})			
		654 nm	541 nm	522 nm	408 nm
**This work**	(40 × 25)	393 ± 11 s	231 ± 4 s	208 ± 4 s	208 ± 4 s
Ref 1[4]	(1400 × 300)	443	194	NR	NR
Ref 2*[5]	31 (diameter)	240 ± 5	200 ± 5 s	NR	NR
Ref 2*[5]	NR	NR	45 (diameter)	410 ± 5	300 ± 5 s

Table S6: Lifetime values (τ) for UCNPs synthesized via thermal decomposition in this work and from literature references. Values are in microseconds (μs). NR: Not Reported. * Values estimated from Figure 3 of Ref 2. The authors do not specify exact values for λ_{em} in the red range. The green wavelength was reported for $\lambda_{em} = 540$ nm, but neither for 522 nm nor the blue range.

References

[1] C. Sorbello, P. Gross, C. A. Strassert, M. Jobbágy and B. C. Barja, *ChemPhysChem*, 2017, **18**, 1407–1414.

[2] A. Casillas-Rubio, D. Mendez-Gonzalez, M. Laurenti, J. Rubio-Retama, O. G. Calderón and S. Melle, **16**, 12184–12195.

[3] J. M. Bujjamer, M. Claudia Marchi, B. C. Barja and H. E. Grecco, *Energy Reports*, 2020, **6**, 63–69.

[4] H. Qin, D. Wu, J. Sathian, X. Xie, M. Ryan and F. Xie, *Scientific Reports*, 2018, **8**, 12683.

[5] J. Zhao, Z. Lu, Y. Yin, C. McRae, J. A. Piper, J. M. Dawes, D. Jin and E. M. Goldys, *Nanoscale*, 2013, **5**, 944–952.
